# A novel tumor-activated ALA fusion protein for specific inhibition on the growth and invasion of breast cancer cells MDA-MB-231

**DOI:** 10.1080/10717544.2017.1406560

**Published:** 2017-11-24

**Authors:** Xiufeng Liu, Xintong Liu, Suwen Sunchen, Meixia Liu, Chen Shen, Juanjuan Wu, Wanli Zhao, Boyang Yu, Jihua Liu

**Affiliations:** a State Key Laboratory of Natural Medicines, China Pharmaceutical University, Nanjing, PR China;; b Jiangsu Key Laboratory of TCM Evaluation and Translational Research, Department of Biotechnology of TCM, China Pharmaceutical University, Nanjing, PR China;; c School of Life Science and Technology, China Pharmaceutical University, Nanjing, PR China

**Keywords:** Fusion protein, tumor activate, uPA, AGAP, apoptosis

## Abstract

**Objective:** The aim of this research was to develop a novel ALA fusion protein for target to the malignant cells surface with high uPAR expression and locally release of the scorpion toxin AGAP in an uPA-cleavable manner. It will provide an effective approach for controlled release of the peptide toxins to treat cancerous cells.

**Methods:** The ALA fusion proteins were expressed in pichia pastoris, and the recombinant proteins were purified by Ni-NTA affinity chromatography. The proteins were added to human breast cancer cells (MDA-MB-231) and human embryonic kidney cells (HEK-293) in order to investigate the characteristic of selective targeting and releasing of scorpion toxin AGAP in cancer cells with high uPAR expression. The inhibitory effect of ALA on MDA-MB-231, MCF7, LO2 and HEK-293 was evaluated by MTT assay. Moreover, the antiproliferation mechanism of ALA was determined by flow cytometric and western blot analysis.

**Results:** The results showed that ALA could target MDA-MB-231 cells and the scorpion toxin AGAP could be released with high efficiency and selectivity. ALA inhibited the growth and invasion of breast cancer cells MDA-MB231. Also, cell apoptosis pathway was found to be associated with the inhibition mechanism of ALA according to the data of flow cytometric and western blot analysis. Therefore, ALA could be a novel antitumor candidate for targeting treatment of malignant cell.

**Conclusions:** This study successfully demonstrated that fusion of biotoxins with tumor target domain could provide a simple yet effective way to delivery of peptide or protein drugs.

## Introduction

Cancer is one of the leading causes of death worldwide (Torre et al., 2015; Siegel et al., 2016). It is estimated that there will be 26 million new cancer cases and 17 million cancer deaths per year by 2030 (Parkin, 2001). To date, chemotherapy remains one of the main options to treat cancer, since chemotherapy can effectively control tumor progression and prolong human life. However, chemotherapy can considerably damage normal cells and increase the risk of the emergence of drug-resistant tumor cells seriously, which limit the effectiveness of available chemotherapeutics. Therefore, there is an urgent need to design more specific and selective anticancer drugs.

Urokinase plasminogen activator uPA and its receptor uPAR are overexpressed in several malignant tumors (Montuori & Ragno, 2014) and could also be served as prognostic markers in cancer (Roy & Walsh, 2014; McMahon & Kwaan, 2015). uPA can bind to cell surface uPAR, thereby activating circulating plasminogen protease which is closely related to adhesion, migration, invasion and metastasis of cancer cells. Various strategies have been applied to target the uPA/uPAR system for cancer therapy. Among them, the amino-terminal fragment (ATF) of uPA which includes the uPAR-binding region but completely lacks the catalytic domain of uPA has been used to design fusion proteins. Supplementary Table S1 shows that the anti-tumor toxins have been fused to ATF for target delivery to cancer cell. The ATF domain in the fusion protein could target delivery anticancer protein or peptide to the cell surface with high uPAR expression level. However, the activation of tumor specification and local release is still a challenge for drug delivery (Andresen et al., 2005).

Scorpion venoms as a potential source of new drug, especially in the anticancer therapeutic have attracted more and more attention recently (Ding et al., 2014; Ortiz et al., 2015). Scorpion venoms and toxins can inhibit cancer growth, induce apoptosis and inhibit cancer progression and metastasis *in vitro* and *in vivo*. Analgesic-antitumor peptide (AGAP) is one of the scorpion toxin polypeptides, consists of 66 amino acid residues, which has been found from the venom of B. *martensii* Karsch (Shao et al., 2007) and has been reported that AGAP can induce apoptosis, cell cycle arrest and growth inhibition in different tumor cells. For example, AGAP suppresses the growth of S-180 fibrosarcoma efficiently and markedly prolongs the survival of mice with engrafted Ehrlich ascites tumor cells (Guo et al., 2016). Also, rAGAP inhibits the proliferation and migration of SHG44 cells by arresting the cell cycle and interfering with cell signal pathways (Zhao et al., 2011). These accumulated reports indicate that the AGAP peptide could potentially be used for cancer therapy. However, the use of peptides as therapeutic agents is hampered by their rapid elimination from the circulation, and accumulation in non-targeted organs and tissues (Bruno et al., 2013; Ezan, 2013; Du & Stenzel, 2014). Moreover, the distribution of anticancer drugs in healthy organs or tissues is highly undesirable because of the potential for severe side effects. Coupled with tumor targeting molecules, including antibodies, peptides, lectins, folate and some vitamins, have been adapted for the delivery of anticancer peptides (Otvos Jr & Wade, 2014; Banga, 2015; Kovalainen et al., 2015). Sometimes, however, the activity of the peptide is attenuated by coupling with the target molecule. Therefore, approaches that are able to release coupled peptides in tumor tissues are supposed to be developed as anticancer therapy.

In the present study, a recombinant eukaryotic expression plasmid containing the ATF and AGAP fusion gene linked with the uPA-cleavable linker gene was constructed and the ALA fusion protein was successfully expressed and purified. The result indicated that ALA fusion protein was able to target to MDA-MB-231 cells and the scorpion toxin AGAP could be locally released with high efficiency and selectivity. Also, ALA inhibits the proliferation, invasion and induces the apoptosis of MDA-MB-231 cells. The ALA fusion protein may be a potential agent for cancer therapy, therefore, it is important for future studies.

## Materials and methods

### Reagents and materials

Restriction enzymes (EcoR1 and Xba1), T4 DNA ligase, DNA marker were purchased from Takara (Dalian, China). The protein marker was purchased from Thermo Fermentas (Guangzhou, China). *E. coli* DH5α, *Pichia pastoris* GS115 pPICZαA vector were stored by our laboratory. Zeocin antibiotic was obtained from Invitrogen (Carlsbad, CA). Anti-Bcl2, anti-Bax, anti-GAPDH, anti-Myc, HRP-goat anti-rabbit conjugate were purchased from Cell Signaling Technology (Danvers, MA). HEK-293 human primary embryonic kidney cells, human breast cancer cell MDA-MB-231 and MCF-7, human liver cell LO2, human liver cancer cell SW460, human glioma cell SHG-44 and human cholangiocellular carcinoma cell Hucc-T1 were obtained from the American Type Culture Collection. All the other chemicals were of analytical grade. Annexin V-FITC apoptosis detection kit was a product of BD Biosciences, USA.

### Construction of pPICZαA-*ALA* plasmid

**Figure 1.**
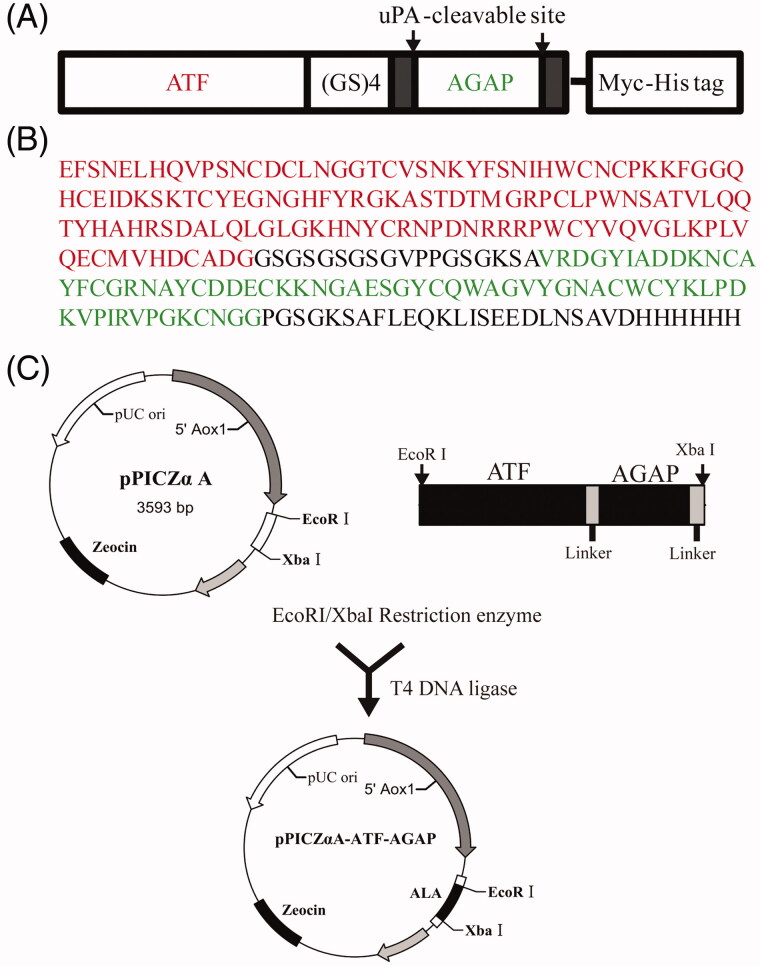
Recombinant expression strategy of ALA fusion protein. (A) Schematic representation of ALA fusion protein. The fusion protein ALA includes the ATF domain, the AGAP peptide, a (GS)4 linker, two uPAcleavable sites and a C-terminal Myc-tag and His6-tag. (B) Full amino acid sequence of ALA. The ATF sequence is marked in red; the AGAP sequence is marked in green. (C) Schematic map of the construction of the recombinant expression vector pPICZαA-ALA.

Based on the amino acid sequence of ATF, uPA cleavable linker and AGAP (Figure 1(B)), the coding sequence was optimized according to the codon bias of *P. pastoris* (http://www.kazusa.or.jp/codon). The optimized nucleotides were synthesized by Jinweizhi Biological Technology Co., Ltd. (Suzhou, China). The DNA of ALA was inserted into the EcoR I and Xba I sites in pPICZαA vector in order to obtain the plasmid pPICZαA-*ALA* (Figure 1(C)). The DNA sequences of the constructed plasmids were confirmed by DNA sequencing by Jinsirui Biotechnology Co., Ltd, (Nanjing, China).

### Expression and purification of ALA

The recombinant plasmid pPICZαA-*ALA* was transformed in to the *P. pastoris* strain GS115 by electroporation. The resultant transformants are screened and optimized for the expression of the recombinant ALA protein. To obtain the ALA protein, the recombinant strain containing the plasmids were cultured in the BMGY medium at 28 °C, 250 rpm shaking incubator. After 24-h incubation, the yeast solutions were reached to an OD600 of 6.0, the cells were harvested by centrifugation, redissolved in BMMY medium and then induced by methanol with a final concentration of 1.0% to express the fusion proteins at 26 °C. After induced for 24 h, the supernatant were collected by centrifugation with 2000 rpm for 10 min at 4 °C. The proteins were purified by Ni-NTA resin column (Jinsirui Biotechnology Co., Ltd, Nanjing, China) and then analyzed by SDS-PAGE and Western blot analysis. The protein concentrations were measured using the BCA protein assay kit (Pierce).

### SDS-PAGE and western blot analysis

Samples were separated by SDS-PAGE in 10–16% gels and stained with 0.05% coomassie brilliant blue or transferred to a poly-vinylidene difuoride (PVDF) membrane (Millipore) using a electroblotting apparatus (Bio-Rad Laboratories, California) at 200 mA for 1 h. The membrane was blocked with 5% skim milk for 1.5 h at room temperature. Then, the membrane was incubated with primary antibodies against for 24 h at 4 °C. After washing, the membrane was incubated with HRP conjugated second antibody for 1 h. The target proteins were visualized and imaged by the Gel Doc XR System (Bio-Rad, Hercules, CA).

### Cell culture

MCF-7, MDA-MB-231, HEK-293 and LO2 cells were cultured in Dulbecco’s modified Eagle medium (DMEM) containing 10% fetal calf serum (FBS), 100 u/ml penicillin, and 100 mg/ml streptomycin antibiotics. Both cell lines were maintained in humidified 5% CO_2_, and incubated at 37 °C in an incubator (Thermo Fisher Scientific, Waltham, MA). 

### Cell viability assay

Effect of toxins on cell viability was measured by 3-(4,-5-dimethylthiazo-2-yl)-2, 5-diphenyltetrazolium bromide (MTT) assay. Cells were seeded onto the 96-well plates at a density of 5 × 10^3^ cells/well and incubated overnight. Different concentrations of toxins were added into the cell media. After incubated for 48 h, the 20 ml MTT solution (5 mg/ml) was added and incubated at 37 °C and 5% CO_2_ for another 6 h. Subsequently, the media were removed from the cells and 150 ml DMSO was added into each well to dissolve the formazan crystals. Absorbance at 570 nm was detected on a micro-plate reader (Bio-Rad) with a reference at 630 nm serving as a blank. The influence of the fusion protein on cell activity was evaluated and compared with control. The control cells were set to 100% activity. The mean value of five wells was counted. Each group was repeated three times.

### Cell apoptosis assay

MDA-MB-231 cells were preseeded at a concentration of 5 × 10^5^ cells per well into 6-well plates and cultured at 37 °C in 5% CO_2_ humidified atmosphere for 24 h. ALA or AGAP were added to the cell culture at a final concentration of 0.3 uM. After 36-h incubation, the cells were harvested and treated with the Annexin V-FITC apoptosis detection kit according to the suggested procedure. The apoptotic cells were detected by flow cytometry.

### Cell invasion assay

Invasion assay was performed by using 24-well transwell units (Corning) with polycarbonate filters (8 μm pore size) each filter was precoated with 100 μl of 1:20 (v:v) diluted matrigel (BD Biosciences, Billerica, MA) in cold medium to form a thin continuous film on the upper chamber. MDA-MB-231 cells were suspended in serum-free medium and placed on the upper chamber and then treated with 0.3 uM AGAP or ALA. The lower chambers were added culture medium containing 10% FBS. After 24-h incubation, cells on the upper chamber were completely wiped away using cotton swabs. Cells on the lower chamber were fixed with methanol for 10 min, stained with crystal violet and then counted under a microscope.

### Statistical analysis

Data were expressed as mean ± SD. Statistically significant differences among groups were determined by one-way ANOVA with adopting the Tukey’s multiple comparison test as the *post hoc* test (GraphPad, San Diego, CA). Data with *p* values less than .05 were considered statistically significant.

## Results

### Design and construction of ALA expression system

A fusion protein ALA consisting of ATF and AGAP which are connected to each other by the (GlySer)4 inker peptide was designed in our study. Two uPA-cleavable sites and a C-terminal Myc-tag and Histidine-6 (His6) tag are included in the fusion protein ALA (Figure 1 (A,B)). The yeast codon optimized *ALA* genes were synthesized and digested by EcoRI and XbaI subsequent inserted into the backbone of the plasmid pPICZαA (Figure 1(C)). The recombiant plasmid pPICZαA-*ALA* was confirmed by DNA sequencing and transformed into the host strain *P. pastoris* GS115. The positive transformants were selected on YPD plates containing 500 µg/ml zeocine. The resulting transformants were further confirmed by PCR analysis of genomic DNA demonstrated that the gene of ALA was integrated into the stable transformants successfully.

### Expression and purification of ALA

**Figure 2.**
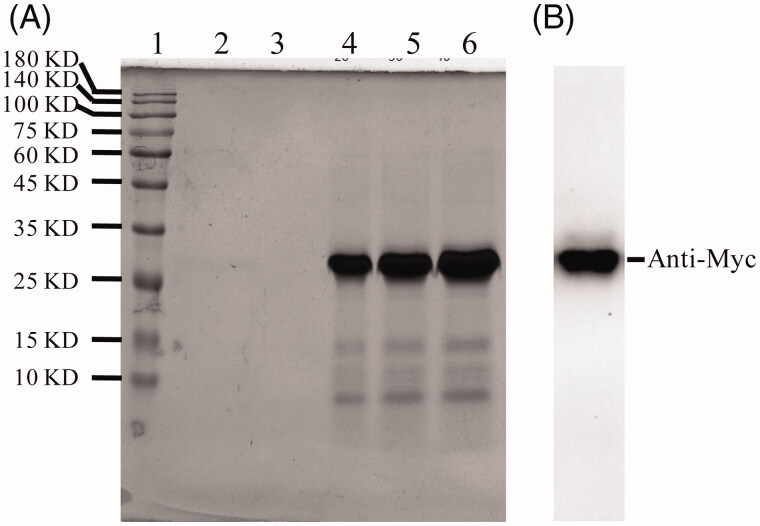
Expression and purification of ALA. (A) 12% SDS-PAGE analysis of the purification of fusion protein by NI-NTA affinity chromatography. Lane 1, protein molecular weight marker; Lane 2, supernatant of the ALA transformants after induction by 1.0% methanol for 24 h; Lane 3, supernatant postbiding to Ni-NTA resin; Lane 4-6, imidazole eluted fraction. (B) Western blotting analysis of ALA with mouse anti-Myc antibody.

One of the *P. pastoris* transformants with the highest expression level of ALA was selected for scaled-up protein production. The *P. Pastoris* strain was cultured and induced with 1.0% methanol in BMMY media. After 48 h of culture, the medium was harvested by centrifugation and the supernatant was purified by Ni-NTA affinity chromatography and analyzed by SDS-PAGE followed by staining with Coomassie Brilliant Blue. The purity of ALA was approximately 95%. The molecular weight was determined to be about 27 KDa (Figure 2(A)), which is consistent with the theoretical value. The primary purified recombinant ALA protein was identified by western blotting which demonstrated that the 27 KDa band was identified by anti-Myc antibody (Figure 2(B)).

### Binding of ALA to breast cancer MDA-MB-231 cells

**Figure 3.**
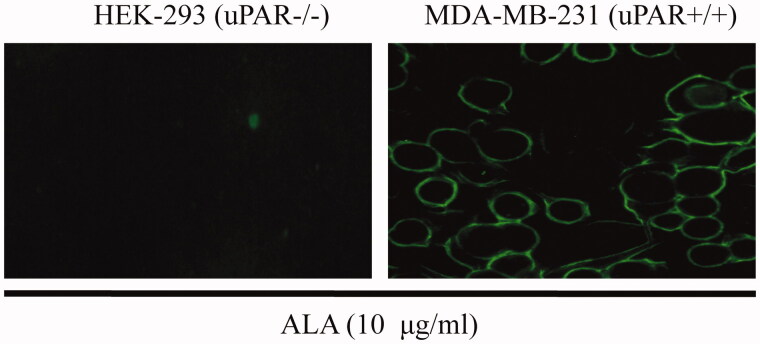
ALA selective binding to MDA-MB231 cells. HEK-293 or MDA-MB231 cells were treated with 10 ug/ml ALA for 4 h and then fixed in 4% paraformaldehyde. The ALA was detected by using an anti-Myc antibody and fluorescein-labeled secondary antibody. Green fluorescence shows ALA fusion protein exhibited specific affinity to MDA-MB-231 cells and mainly located on the surface.

To determine whether the fused AGAP influences the ability of ALA fusion proteins to target tumor cells, the uPAR expression is critical for the binding of ALA to cell surface. An immunofluorescence assay was performed against breast cancer MDA-MB-231 cells (expressed high levels of uPAR) and uPAR-negative HEK-293 epithelial cells. Purified ALA proteins were added to cultured MDA-MB-231 or HEK-293 cells for 4 h of incubation. As shown in Figure 3, the ALA fusion proteins can bind to the surface of MDA-MB-231 cells but not HEK-293 cells, indicating ATF helped the fusion proteins to target to cell surface in living cells as expected.

### *In vitro* selective anticancer effects of ALA

**Figure 4.**
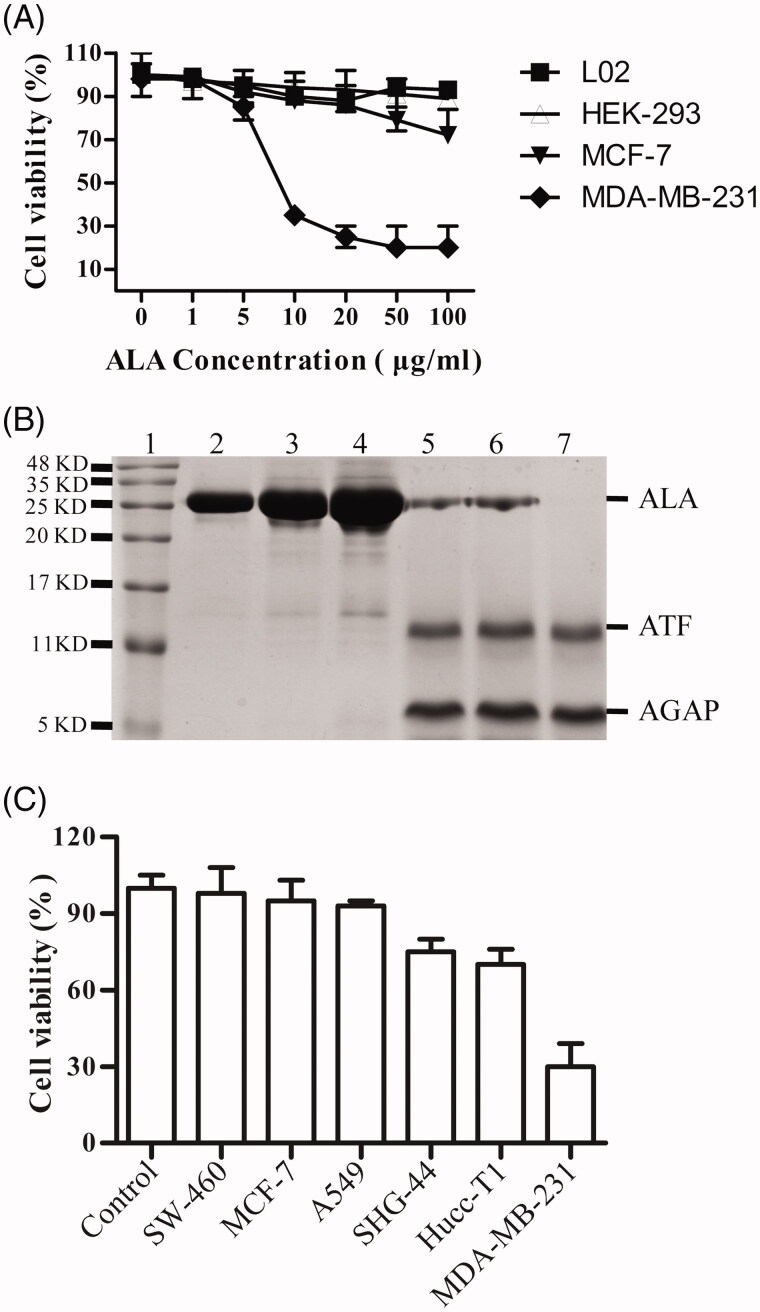
Tumor-selective activation of ALA. (A) The growth curves of different cell types treated by ALA. The human breast cancer cell lines MDA-MB-231 and MCF-7, human embryonic kidney cell line HEK-293 and normal human liver cell line L02 were treated with the indicated concentrations of ALA for 48 h, and then, the cell viability was measured by MTT assay. (B) 16%Tris-tricine SDS-PAGE analysis of the ALA cleavage and release of AGAP. Lane 1, protein molecular weight marker; Lane 2-4, 5 ug/ml, 10 ug/ml and 20 ug/ml ALA added to HEK-293 cell culture medium for 24 h; Lane 5-7, 5 ug/ml ALA incubated with MDA-MB-231 cell culture medium for 12 h, 24 h and 36 h, respectively. (C) The inhibition effects of ALA on different cancer cells. Cells were treated with 10 ug/ml ALA for 48 h, and then, the cell viability was measured by MTT assay.

The ability of ALA inhibit proliferation of different cell lines was compared. As shown in Figure 4(A), the MTT assay indicated that the fusion protein ALA influenced the proliferation of breast cancer cell lines MDA-MB-231 more significantly than that of MCF-7 cells. The proliferation of the MDA-MB-231 cells significantly decreased in a dose-dependent manner and only slightly inhibited in MCF-7 cells proliferation at the higher dose of 50–100 ug/ml. However, the effects of ALA on the viability of HEK-293 cells and LO2 cells were not observed with increased concentration of ALA. In order to determine whether the ALA fusion protein can be cleaved by the cell secreted uPA and releases of the AGAP peptide, ALA proteins were added to HEK-293 (non-expressing of uPA) or MDA-MB-231 cells at different concentrations and time points, then the proteins were detected by SDS-PAGE assay. As shown in Figure 4(B) (Lane2-4), there was no obvious digest of ALA when incubated with the cultured HEK-293 cells for 24 h. The fusion protein ALA was stable in the uPA-negative cells. However, the ALA can be cleaved when incubating with MDA-MB-231cells for 12 h, 36 h or 48 h, with the appearance of two bans at 11-16 KD and 5-11 KD (Figure 4(B), Lane 5-7). It is consistent with the predicted 16.3 KD and 7.3 KD, corresponding to the ATF fragment and AGAP peptide, respectively. These data demonstrate that the ALA could be selectively activated and inhibit the proliferation of cancer cell with high expression level of uPAR and uPA.

### Effects of ALA on MDA-MB-231 cell apoptosis

**Figure 5.**
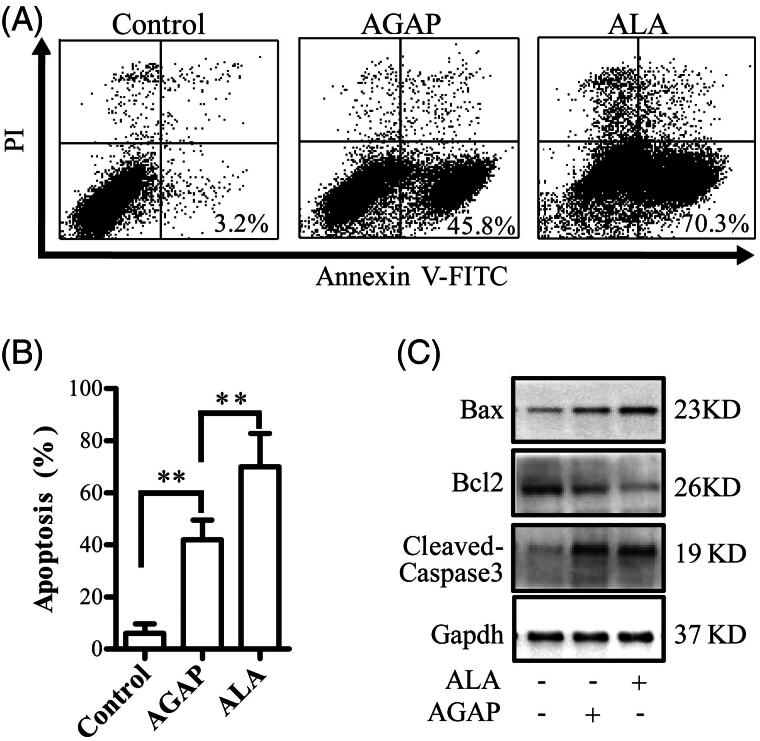
Effects of ALA on cell apoptosis in MDA-MB-231 cells. (A) Breast cancer cells MDA-MB-231 were treated with 0.3 uM ALA or AGAP for 36 h, and then, the apoptotic cells as percentages of total cells measured by AnnV/PI fluorescence staining and flow cytometric analysis. (B) Quantification of the apoptotic rate in (A). (C) Expression of apoptosis regulatory proteins, bax, bcl-2 and cleaved-caspase3, were determined using Western blot analysis. GAPDH levels are used as loading controls. Data are expressed as the mean ± S.D. of three experiments. *p < .05 indicates statistically significant differences from control cells.

The apoptosis-inducing effect of AGAP and ALA in MDA-MB-231 cells was determined using a flow cytometric analysis and the number of apoptotic cells was quantified. As shown in Figure 5(A,B), both AGAP and its fusion protein ALA-induced apoptosis in the MDA-MB-231 cells, compared to the untreated-controls. However, ALA triggers a higher degree of apoptosis than AGAP by the same concentration at 24 h. MDA-MB-231 cells were exposed to 0.3 uM AGAP for 36 h, the apoptosis rate was 45.75%. In contrast, ALA showed a much higher apoptosis rate of 70.28%. To explore the mechanism underlying the better apoptosis inducing effects by ALA, the effects of ALA or AGAP treatment on the expression levels of Bcl-2, Bax and the activation of caspase 3 were examined in MDA-MB-231 cells using Western blotting assay. As shown in Figure 5(C), after treated with ALA for 36 h, the expressions of pro-apoptotic proteins cleaved-caspases 3 and Bax increased, but Bcl-2 decreased compared with AGAP group (Figure 5(C)).

### Effect of ALA on MDA-MB-231 cell invasion

**Figure 6.**
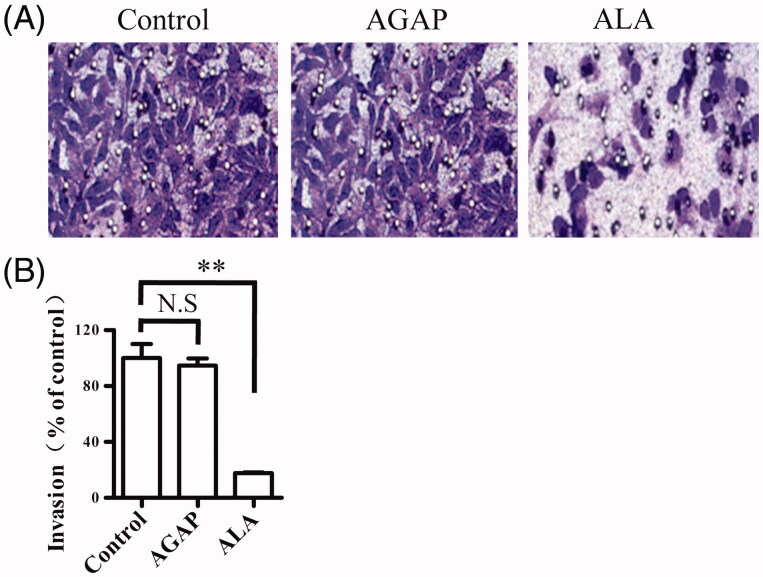
Effects of ALA on migration in breast cancer cells MDA-MB-231. (A) Representative micrographs of transwell migration assay. The cells passing through the matrigel basement membrane of transwell chambers were stained by crystal violet, and the staining cells represent the invasion ability of cells. (B) Quantification of cell migration expressed as the percentage of control. Data are expressed as the mean ± S.D. of three experiments. **p < .01 indicates statistically significant differences from control cells. N.S, no significance.

It has been validated that ATF fragment not only target tumor cell but also inhibit tumor cell invasion. We next assessed whether ALA could affect the ability of breast cancer cells to invade using a transwell assay. MDA-MB-231 treated with 0.3 uM AGAP or ALA in the trans-well chamber for 24 h, crystal violet staining was used to detect the number of cells to pass through the matrigel membrane. As shown in Figure 6, the invasion of the cells treated with ALA was significantly inhibited compared with that in the untreated or AGAP-treated cells. This result suggests that ALA could suppress MDA-MB-231 invasion *in vitro*.

## Discussion

Biotoxin, such as AGAP peptide from the scorpion venom, is a promising anti-cancer drug candidate (Ding et al., 2014; Ortiz et al., 2015). However, there is a formidable challenge for these small peptides to be used in clinical, including fast elimination from the systemic circulation by enzymatic degradation or renal filtration, accumulation in nontargeted tissues (Torchilin & Lukyanov, 2003). ATF is the amino-terminal fragment of uPA, which includes the uPAR-binding region but completely lacks the catalytic domain of uPA. uPA and its receptor uPAR are over-expressed in a wide range of invasive tumor cells, but rarely expressed in normal quiescent tissue, therefore they are believed to play a critical role in tumor invation, migration and adhesion (Roy & Walsh, 2014; McMahon & Kwaan, 2015). To date, there have been accumulating evidences that ATF is a powerful drug carrier for anti-cancer drug delivery (Todhunter et al., 2004; Takei et al., 2005; Hall & Vallera, 2006; Sun et al., 2008; Provenzano et al., 2016; Su et al., 2016), with a dissociation constant of 0.2 nM to the cell surface uPAR (Li et al., 2014). Various cargos have been delivered by ATF (e.g. genes, proteins, peptides and nanoparticles, etc.) directly to the tumor tissues. As AGAP requires a powerful carrier and protector, while ATF is in need of a carrier that facilitates cancer cell targeting and protects from enzymatic degradation and renal filtration, fuzing AGAP and ATF appeared to be effective method, to achieve the goal.

In the present study, a fusion protein named ALA contains the ATF domain, AGAP peptide and an Upa-cleavable linker between the fused domains was designed (Figure 1(A)). The ATF domain is considered to deliver the AGAP into tumor tissues. Once the ALA protein was delivered to tumor tissues, the AGAP can be released into the environment of tumor by uPA specific cleave of the linker. Several ATF or ATF fusion proteins were expressed in *E. coli* systems by other groups and the recombinant proteins seem easy to accumulate as insoluble inclusion bodies (Hall & Vallera, 2006; Sun et al., 2008). This may be caused by misfolding of the cysteine-rich ATF in the bacteria. Thus, a yeast recombinant expression system was closed to expression of the fusion protein ALA in this study. Our results showed that the ALA proteins were successfully expressed in *P. pastoris GS115* in soluble form and the molecular weight was determined to be about 27 KDa (Figure 2(A)), which is consistent with the theoretical value.

After successful purification of the ALA fusion proteins, the function assay was conducted. ALA proteins were incubated with the uPAR-positive MDA-MB-231 or uPAR-negative HEK-293 cells for 4 h. The immunofluorescence results showed that ALA can bind to the cell surface of MDA-MB-231cells, but not to that of HEK 293 cells. This result clearly proved that the function of ATF domain was not affected by fusion with AGAP. In accordance with others studies, the ATF is demonstrated to be a powerful drug carrier for anti-cancer drug delivery (Hansen et al., 2013; O’Halloran et al., 2013). Our results also revealed that ALA fusion protein exhibited significant toxicity to MDA-MB-231 breast cancer cells in a dose-dependent manner, only slight toxicity to MCF-7 breast cancer cells at the higher dose of 50–100 ug/ml and nontoxic to normal HEK-293 and LO2 cell lines, making ALA to be a promising candidate for anticancer drug developing. The growth of two breast cancer cell lines, MDA-MB-231 and MCF-7, that suffered different inhibition effects, may be caused by the differences in the uPA expression between two cell lines. uPA is secreted to the culture medium and digested the cleavable linker to release free AGAP. It has been reported that MCF-7 cells had low levels of uPA compared with MDA-MB-231 cells (Holst-Hansen et al., 1996). Subsequently, in order to determine whether the fusion protein ALA could be cleaved to release the AGAP domain, the ALA proteins were added to HEK-293 and MDA-MB-231 cell culture medium, respectively. Our results showed that ALA was stable in the HEK-293 cell-cultured medium for 24 h. However, The ALA could be cleaved when incubating with MDA-MB-231 cell culture medium for 12 h, with the appearance of two bans at 11-16 KD and 5-11 KD (Figure 4(B), Lane 5-7). It is consistent with the predicted 16.3 KD and 7.3 KD, corresponding to the ATF fragment and AGAP peptide. These effects were mediated by uPA, since HEK-293 cells lacking uPA secretion and the fusion proteins were not digested.

AGAP has been found to inhibit the proliferation of various tumor cells. The mechanism of cytotoxicity of the AGAP is not yet very clear, AGAP can inhibit the tumor cells by various ways including up-regulation of the expression of pro-apoptotic proteins, down-regulation of the expression of anti-apoptotic proteins for induction apoptosis, and arrestion of cell cycle at G1 phase (Shao et al., 2007; Gu et al., 2013; Guo et al., 2016). In this study, the apoptosis rate were detected by flow cytometry to determine the anticancer mechanism of ALA, MDA-MB-231 cells treated with 0.3 uM ALA or rAGAP for 36 h. The total percentage of apoptotic cells in AGAP and ALA treatment groups were 45.8% and 73.0%, respectively. The apoptosis rate of MDA-MB-231 cells treated with ALA was relatively higher than that of AGAP (Figure 5(B)). These data indicated that ATF as a carrier does not influence the apoptosis function of AGAP and the inhibition mechanism on cell growth of ALA might be related to the induction of cell apoptosis.

## Conclusions

The study suggested that ATF acted as a specific cell-targeting molecular was able to deliver the functional peptide AGAP to the cell surface of MDA-MB-231 cells. The fusion protein ALA could be activated by uPA digestion and release free toxin APAP. ALA exhibited obvious inhibition on MDA-MB-231 cell growth and invasion, which might provide a potential and effective approach for protein therapy of malignant tumors with high expression level of uPA and uPAR. Apoptosis was involved in ALA-induced cell death and the mechanism was associated with the change in Bax/Bcl-2 ratio and caspase-3 activity.

## Supplementary Material

IDRD_Liu_et_al_Supplemental_Content.doc
