# Supplementary material for: A novel tumor-activated ALA fusion protein for specific inhibition on the growth and invasion of breast cancer cells MDA-MB-231
Source: Drug Deliv. 2017 Nov 24;24(1):1811–7. doi: 10.1080/10717544.2017.1406560 (PMC8241173; doi:10.1080/10717544.2017.1406560)
Supplement: IDRD_Liu_et_al_Supplemental_Content.doc [file IDRD_A_1406560_SM0352.doc]

**Supplementary Data**

**A novel tumor-activated ALA fusion protein for specific inhibition on the growth and invasion of breast cancer cells MDA-MB-231**

Xiufeng Liu^a,b^, Xintong Liu^c^, Suwen Sunchen^a^, Meixia Liu^a^, Chen Shen^a^, Juanjuan Wu^a^, Wanli Zhao^a^, Boyang Yu^a,b^ and Jihua Liu^a,b^*

^a^ State Key Laboratory of Natural Medicines, China Pharmaceutical University, Nanjing 210009, PR China; ^b^ Jiangsu Key Laboratory of TCM Evaluation and Translational Research, Department of Biotechnology of TCM，China Pharmaceutical University, Nanjing 210009, PR China; ^c^ School of Life Science and Technology, China Pharmaceutical University, Nanjing, P. R. China

*Correspondence to: Ji-Hua Liu; Email: [liujihua@cpu.edu.cn](mailto:liujihua@cpu.edu.cn);

**Supplementary Table 1: An overview of various ATF based recombinant toxins for cancer t**herapy

| **Name** | **Toxin** | **Origin of the Toxin** | **Toxin Cleavage** | **Host** | **Cancer cell** | **Reference** |
| --- | --- | --- | --- | --- | --- | --- |
| DTAT | Diphtheria toxin | Cory-nebacterium diphtheriae | No cleavage | *E. coli*  BL21 (DE3) | U118MG cells | . |
| ATF-SAP | Ribosome inactivating protein saporin | Plant | No cleavage | Pichia  pastoris | U937 leukemia cells |  |
| ATF-  Mellitin, | Melittin | Honey bee | No cleavage | Pichia  pastoris | SKOV3 cells |  |
| ALV | Antiangiogenic domain of vasostatin | Human | No cleavage | *E. coli*  BL21 (DE3) | MDA-MB-231  cells |  |
| ATF-HI8 | Kunitz-type protease inhibitor | Human | No cleavage | HRA cells | ovarian cell line |  |
| DTAT13 | Diphtheria toxin | Cory-nebacterium diphtheriae | No cleavage | *E. coli*  BL21 (DE3) | glioblastoma cell lines | ] |
